# Finding the shortest path with PesCa: a tool for network reconstruction

**DOI:** 10.12688/f1000research.6769.2

**Published:** 2016-04-07

**Authors:** Giovanni Scardoni, Gabriele Tosadori, Sakshi Pratap, Fausto Spoto, Carlo Laudanna

**Affiliations:** 1 Center for Biomedical Computing, University of Verona, Verona, Italy; 2 Birla Institute of Technology & Science, Goa, India; 3 Department of Computer Science, University of Verona, Verona, Italy; 4 Department of Medicine, University of Verona, Verona, Italy

**Keywords:** biological networks, shortest path, pesca, protein protein interaction networks, connect isolated node, cytoscape

## Abstract

Network analysis is of growing interest in several fields ranging from economics to biology. Several methods have been developed to investigate different properties of physical networks abstracted as graphs, including quantification of specific topological properties, contextual data enrichment, simulation of pathway dynamics and visual representation. In this context, the PesCa app for the Cytoscape network analysis environment is specifically designed to help researchers infer and manipulate networks based on the shortest path principle. PesCa offers different algorithms allowing network reconstruction and analysis starting from a list of genes, proteins and in general a set of interconnected nodes. The app is useful in the early stage of network analysis, i.e. to create networks or generate clusters based on shortest path computation, but can also help further investigations and, in general, it is suitable for every situation requiring the connection of a set of nodes that apparently do not share links, such as isolated nodes in sub-networks. Overall, the plugin enhances the ability of discovering interesting and not obvious relations between high dimensional sets of interacting objects.

## Introduction

Network analysis is a hot area of investigation in different, apparently unrelated, research fields. In particular in biology, biotechnology, and biomedical research, consistent efforts are being carried out in order to investigate how complex biological processes work ^1^. In this scenario a disease, a metabolic pathway or a coexpression microarray could be analyzed by means of network theoretical formalism such that the structural properties of the models can be quantified ^2^. The central point of this approach concerns the emergence of peculiar properties ^3^ that arise when a set of distinct objects reciprocally interact generating a functionally integrated system. In this context, the goal is to uncover the complex behaviours, hidden by the system’s complexity, that are specific to that particular process. The interactions between the objects are abstracted as graphs and analyzed by means of graph theory. Thus, it is possible to study the topological role of each network component (node), to uncover hidden structural patterns, find clusters, or even simulate the time evolution of specific network topologies.

In order to identify the hidden properties of a complex system, as a first step it is necessary to construct a network representing the system under investigation. Cytoscape ^4^ has several built-in tools allowing network construction and analysis. Notably, in Systems Biology a common methodology implies that the informational flow follows a maximum parsimony principle (see Box 3 in 5). Consequently, computing the paths in a network can have direct functional implications that affect the topology and the characteristics of the whole network. It must be considered that there are different techniques that permit to retrieve the paths between the nodes, that depend on the context, the process under investigation and the kind of interactions that are modelled. Here we describe PesCa, a novel Cytoscape app specifically designed to compute shortest paths between two or more nodes in a network, thus permitting the construction of sub-networks based on a shortest paths computation. The generated clusters allow focusing the analysis on sets of nodes characterized by reduced topological complexity. Many options are also implemented, enabling the users to investigate different aspects of network complexity.

## Methods

### Implementation and operation

PesCa is a Cytoscape app, thus it is not standalone, but only works in conjunction with the Cytoscape environment. The release of PesCa presented here is developed for the 3.x Cytoscape series. The version for the 2.x series is no longer updated and lacks the features of the new release. Since the new version of Cytoscape has a new structure and uses a different architecture the new PesCa is developed and maintained only for the 3.x platform.

The PesCa core is based on the All Pair Shortest Paths (APSP) version of the basic Dijkstra algorithm ^6^, single thread; it performs a modified version of the APSP search that finds all the shortest paths between each couple of nodes. Furthermore, PesCa offers further options: for example the *Multi Shortest Paths (S-P Cluster)* is an APSP version computing the shortest paths between all selected nodes in a specific network. The *Multi Shortest Paths Tree* is a modified version of an APSP searching all the shortest paths connecting a single selected node to all other nodes in a network. PesCa is also designed to extract a fully connected sub-network from a bigger network, thus allowing connecting nodes that apparently do not interact with a specific set of nodes (here such nodes are called *isolated nodes*).

**Figure 1.**
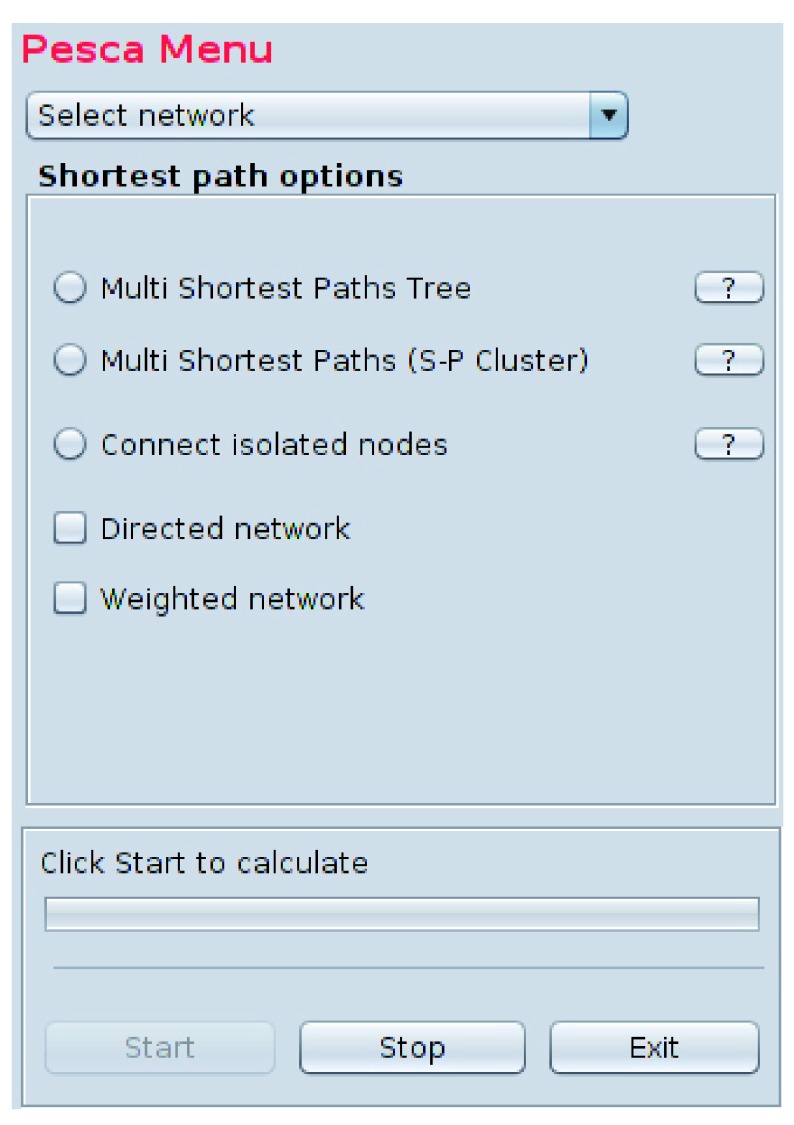
The main panel with all the options PesCa offers.

Figure 1 shows the main panel and the tasks that can be accomplished with PesCa:

• *Multi Shortest Paths Tree* allows computing all the shortest paths connecting a node to all other nodes in a network.
• *Multi Shortest Paths (S-P Cluster)* allows computing the shortest paths connecting two or more selected nodes in the network. It allows generating network sub-clusters (modules) based on minimal cost.
• *Connect isolated nodes* allows finding all the connecting shortest paths between a selected node and a group of other nodes in a network (the so called *Giant component*).

Notably, the *Connect isolated nodes* function connects a node to the nearest nodes in the selected sub-network: this means that the task does not return, as a result, all the shortest paths between the node and the selected sub-network. Only the shortest paths between the node and the nearest node(s) in the selected cluster are given. It is important to note that the nodes that form the selected sub-network don’t have to share links. This sub-network is a set of nodes that is considered as a unique target for this task: the shortest paths are found from the selected node to this set of nodes. Upon selection of the *Connect isolated nodes* function, a wizard dialog opens guiding the user through the sequence of steps necessary to complete the task.

For each function PesCa has a button, indicated with a question mark, that opens a new dialog defining the characteristics and the steps concerning the selected task. The app has several dialogs that appear during usage, designed to help users. For instance, by selecting the *Multi Shortest Paths (S-P Cluster)* option and then clicking on the Start button without choosing the minimum number of nodes required, the app presents a dialog prompting to the user to select two or more nodes. Every time that the selected input does not correspond to the expected input, PesCa presents a dialog in order to help the user in selecting the appropriate entries.

It is also possible to analyze directed and undirected networks, depending on the characteristics of the edges. If a network is directed it can happen that certain nodes cannot be reached. For instance, if a node has an In-Degree equal to zero it cannot be reached while a node that has an Out-Degree equal to zero does not reach other nodes. These two situations can cause some trouble while performing a topological analysis. When a node has a degree or, in the case of directed network, has an Out degree equal to zero, it is not possible to connect it to the network since the information about its interaction is missing. This means that no links are known for that specific node. In this case it is possible to search for more interactions by using, for example, different other datasets, that consider more nodes or edges. It is also possible to use some of the available on-line databases. In any case, if a node is disconnected and no interactions are known, it will be impossible to connect it to the network and it is not possible to infer its topological characteristics.

Analysis of networks with weighted edges is also allowed. Notably, edges can have positive and negative weights. If this option is selected, after clicking *Start*, a dialog will appear asking for the name of the attribute that stores the information about the weights (edge attribute). Weighted edges can simply correspond to edges length but can also provide information about the functional influence of a node on another node(s), such as, for instance, in transcriptomics networks. Thus, this PesCa function may introduce interesting possibilities in the analysis of gene expression networks and other kinds of networks.

With relatively small networks, e.g. the IntegrinActivation_FN.sif pre-loaded network, PesCa does not require high rates of memory nor long computational times; for instance, only a few seconds are necessary to perform a *Multi Shortest Paths Tree* on a Xubuntu 13.10 machine with an Intel®i5, 2.80GHz CPU and 4 GB of RAM. This network has 3091 nodes and 97115 edges and can be automatically loaded within PesCa. Indeed, the PesCa panel also offers a Select network scrollable menu that permits loading a set of pre-loaded biological networks, in different file formats. The networks, that represent different biological processes, are a version of the Human interactome compiled from different databases, a version from BioGrid, a version from PathwayCommons, a directed version which stores information about edges directions and an expanded version with more, different edges. Moreover we have a human interactome which stores information about proteins domains and motifs, a network of pathways and genes, a diseasome in which proteins are linked to diseases and finally a network about signaling proteins involved in integrin activation. A more detailed description of these networks is provided at http://dp.univr.it/~laudanna/LCTST/downloads/index.html. These networks, and many others, are freely available for download from this website. These networks can be customised by adding edge weight and nodes attributes.

## Use cases

**Figure 2.**
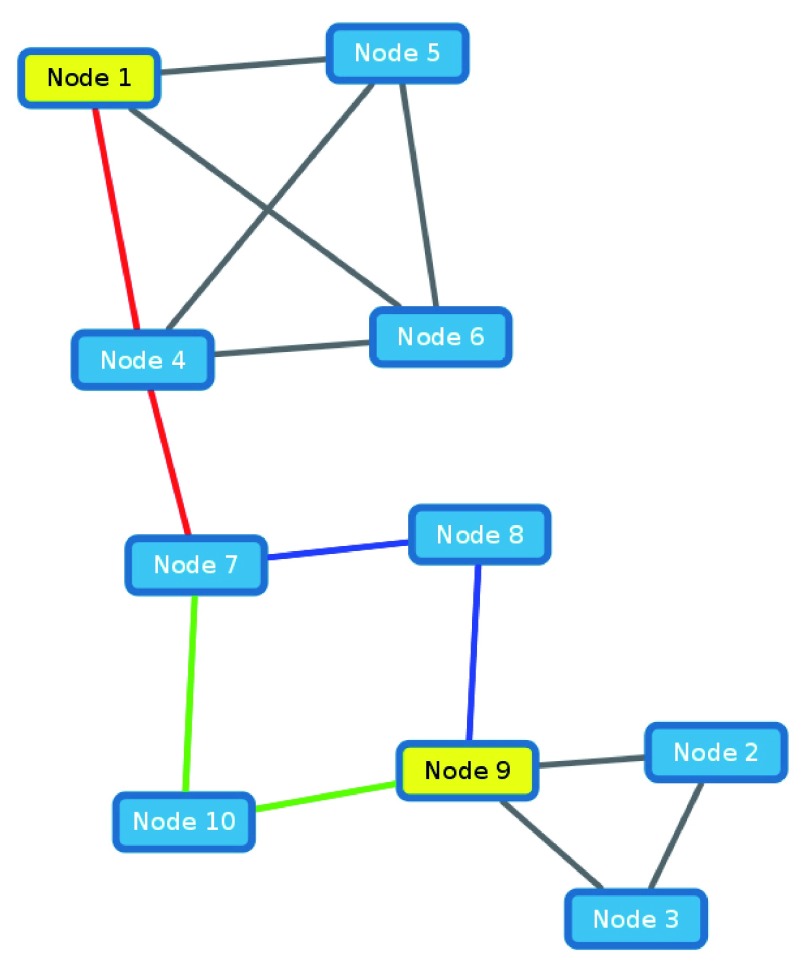
The network we used to perform the *Multi Shortest Paths* retrieval. We used Node 1 as the source and Node 9 as the target. In red the edges that are shared between the two shortest paths. In blue and green the two different, but equivalent, ways for reaching Node 9.

A few case studies are provided, illustrating the functionality of PesCa. The first example describes how to perform a *Multi Shortest Paths (S-P Cluster)* retrieval: the goal is to find all the shortest paths that link two, or more, selected nodes. Figure 2 shows the analysed network (which is provided as Supplementary Material): ten numbered nodes and 14 undirected edges. The nodes that were used to compute the shortest paths are in yellow: Node 1 and Node 9. After node selection, by clicking the *Start* button PesCa performed the search.

**Figure 3.**
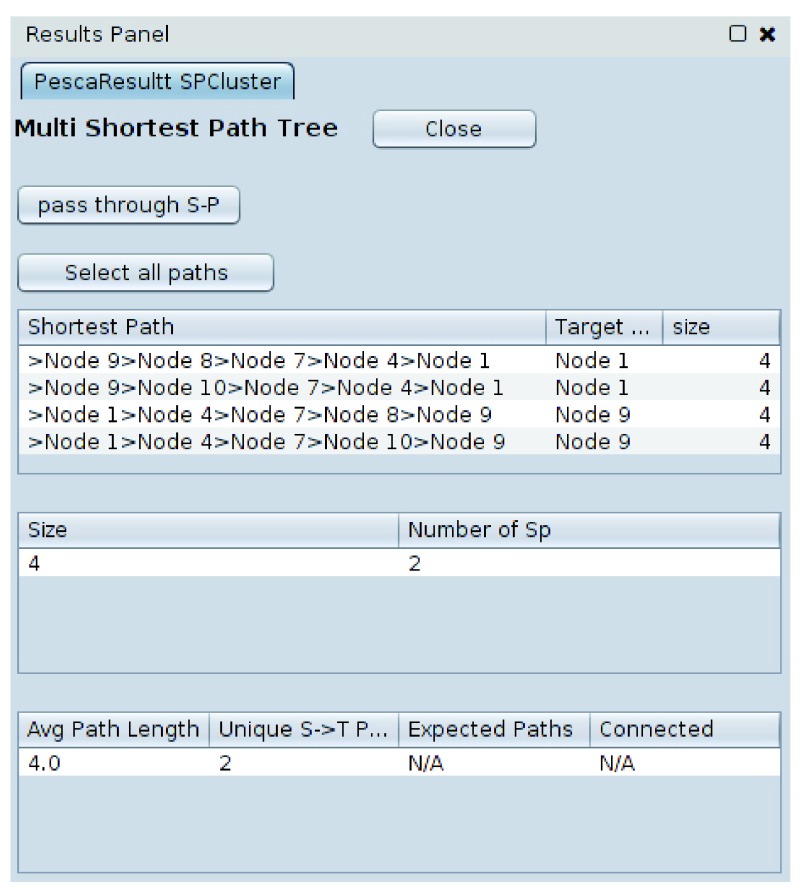
The results panel that shows the result of our analysis with the *Multi Shortest Paths (S-P Cluster)* option.

The result panel in Figure 3 shows the output. The table on the top of the panel lists the retrieved shortest paths, the source for each path and its size. The size, i.e. the length, of a shortest paths is given in terms of how many edges are needed to reach the target. PesCa found four shortest paths, two starting from Node 1 to Node 9 and two starting from Node 9 to Node 1: their length is four. The table below the one already described shows how many paths have a specific length: it groups the paths by their size. In this example PesCa found two shortest paths of length four. It states two shortest paths because the network is undirected and, since the edges are bidirectional, PesCa considers the path “Node 1 to Node 9” equal to the path “Node 9 to Node 1”. Consequently only two paths are listed: one passes through Node 8, 7 and 4, the other one passes through the Node 8, 10 and 4. The last table, at the bottom, shows some characteristics of the network: the average path length is four, the number of unique short paths is two and two other parameters are not relevant to this example.

PesCa retrieved the shortest paths giving the sequence of the nodes involved, which could be highlighted by selecting a specific path in the table. Furthermore, the button at the top left corner, pass through S-P, enables the user to highlight the shortest paths passing through a selected node.

**Figure 4.**
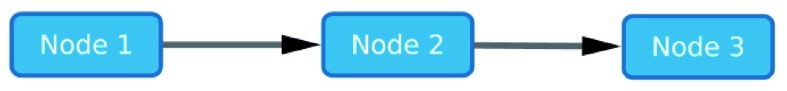
A directed network that has non communicating nodes.

The second example describes the third table in the results panel, the one with the missing values. By using the network in Figure 2 a *Multi Shortest Path Tree* was computed. To carry out this analysis it is necessary to select a node; in this case Node 1 is used. In Figure 7 the results are shown. The interesting point here is the bottom table; all the options are now defined by a value: the average path length, the number of unique shortest paths, the number of expected paths, and the Connected column. The number of expected paths refers to the total number of shortest paths a network is supposed to develop if it is fully connected. The Connected column could be True or False and states if all the nodes are able to communicate together by means of a path. The network in Figure 2 is connected and the column states True. Now, if the edge between Node 4 and Node 7 is removed, then two different connected components will appear. The network is now disconnected and the value will be False. Furthermore, if a network is directed, see Figure 4, the returned value can be both True or False. In the example it will be True if the *Multi Shortest Path Tree* is computed from Node 1. It will be False if the *Multi Shortest Path Tree* is computed from Node 2 and 3 because neither are able to reach Node 1.

**Figure 5.**
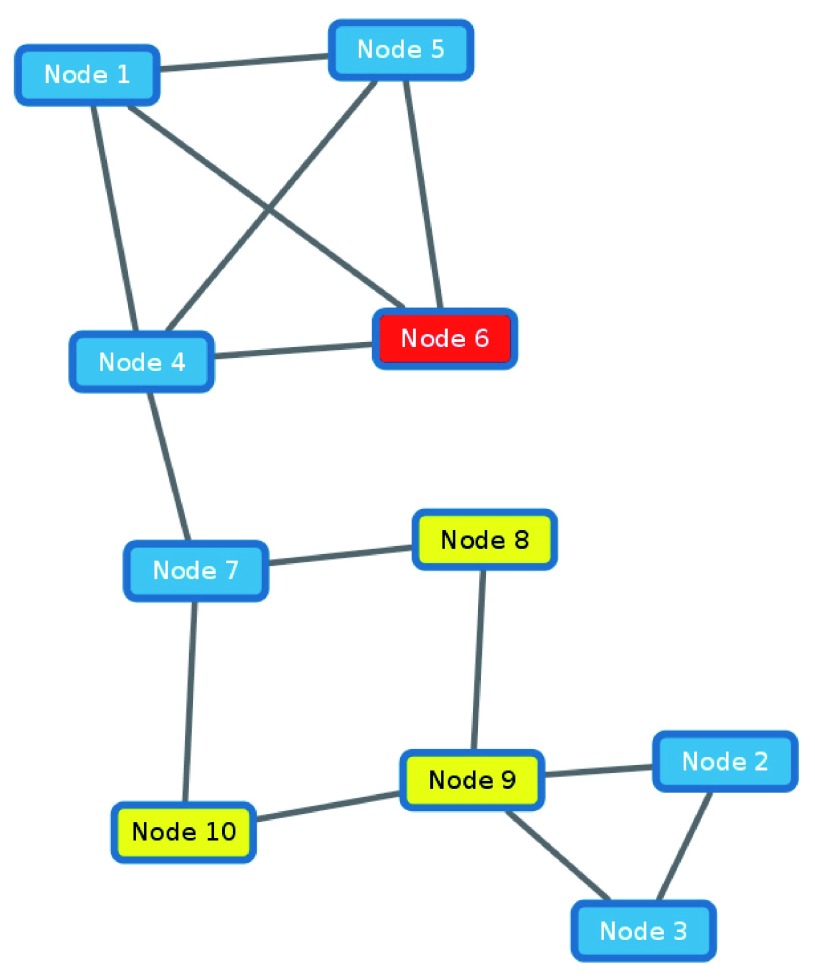
The network we used to show how to connect Node 6 to the sub-network formed by Node 8, 9, 10.

**Figure 6.**
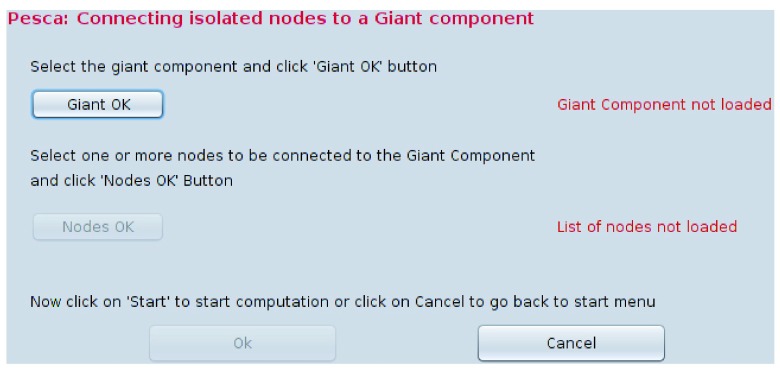
The panel that will help in connecting disconnected nodes.

The third example describes how to use the *Connect isolated component*. Figure 5 shows the network used for the analysis. Again, there are ten nodes, fourteen edges, and a few highlighted nodes in yellow. In this analysis, PesCa retrieved the paths from Node 6 to the cluster formed by Node 8, 9 and 10. After selecting the option, by clicking *Start*, a dialog shows up like the one in Figure 6, and guides the user in selecting the sub-network. This subset is the cluster to which PesCa will connect the node. In this example the component is represented by Node 8, 9 and 10. By selecting it and then clicking *Ok* the user is able to choose the node of interest: highlight Node 6 and then click on *Ok*. Finally by clicking *Start*, PesCa will run the algorithm.

**Figure 7.**
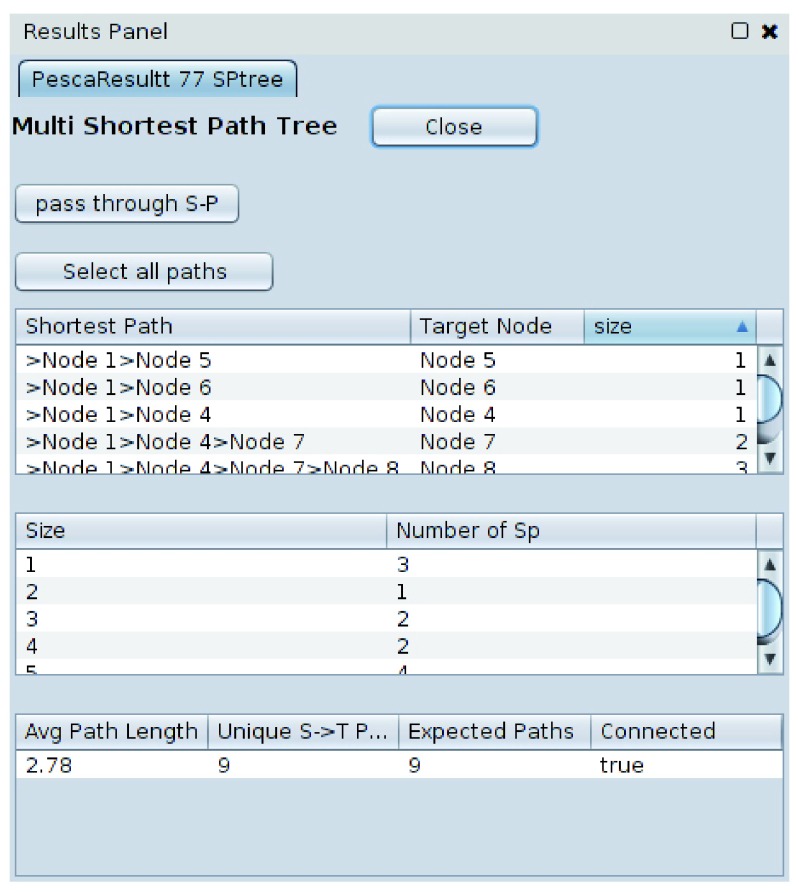
The results panel for the *All nodes Shortest Paths* option.

The results show two shortest paths, one reaching Node 8 and one reaching Node 10; Node 9 is not considered as a target because it does not develop a shortest path with Node 6 since it is connected to Node 6 by means of Node 8 and 10.

## Summary

We have briefly described the main functionalities of PesCa. We described a few application cases by using a very simple network in order to show how to setup the input and how the output panel works. Overall, PesCa is designed for sub-network retrieval and shortest paths search and, in the Cytoscape context, it is the only app that performs this task. It can be used to enhance the predictive power of biological networks by reducing the complexity of the processes under investigation and, in conjunction with other apps, it permits the researcher to deeply investigate the properties of subsets of nodes.

## Software availability

1. Software available from: http://apps.cytoscape.org/apps/pesca30
2. Latest source code: https://bitbucket.org/giovanniscardoni/pescareleaseforcy3public
3. Link to archived source code as at time of publication: http://dx.doi.org/10.5281/zenodo.21145 ^7^
4. Software license: Lesser GNU Public License 3.0: https://www.gnu.org/licenses/lgpl.html

## Supplementary material

Analysed network for use cases.
