# Supplementary material for: Finding the shortest path with PesCa: a tool for network reconstruction
Source: F1000Res. 2016 Apr 7;4:484. Originally published 2015 Aug 5. [Version 2] doi: 10.12688/f1000research.6769.2 (PMC5054806; doi:10.12688/f1000research.6769.2)
Supplement: Peer review history

# Peer review: Referee response for version 2

**DOI:** 10.5256/f1000research.8872.r16563

**Published:** 2016-10-06

**Authors:** Salvatore Alaimo

**Affiliations:** 1 Department of Clinical and Experimental Medicine, University of Catania, Catania, Italy

The authors describe a Cytoscape 3.x plugin, which, on the basis of the shortest path principle, allows researchers to infer and manipulate networks. Their software makes use of a version of the All Pair Shortest Paths of the Dijkstra algorithm in order to infer many characteristics of the network under examination. The application appears to be very useful, and the version 2 of their manuscript seems to be well written. In agreement with the point raised by the other reviewers, I would like to suggest few minor changes to the paper that might improve its readability.

1. Briefly explain some applications of the shortest path principle in systems biology, describing more accurately the maximum parsimony principle and why it is important in biological networks.
2. Put figure 7 before figures 4-5-6 since it is cited before.
3. Add an image that illustrates the results of the "Connected Isolated Nodes" options like 3 or 7.

Regarding the application, I would like to point out one minor change:

- When displaying the "Multi Shortest Path Tree" results on a non-oriented network, you might avoid showing twice the paths composed of the same nodes but in the opposite direction.

I have read this submission. I believe that I have an appropriate level of expertise to confirm that it is of an acceptable scientific standard.

# Peer review: Referee response for version 2

**DOI:** 10.5256/f1000research.8872.r16564

**Published:** 2016-10-04

**Authors:** Anna Zhukova

**Affiliations:** 1 Institut Pasteur, Paris, France

The authors describe a Cytoscape application that performs shortest path calculation in networks. This application is certainly very useful, however a few improvements could be made.

 Changes to the application:

1. Please, add an app description in Cytoscape App Manager.
2. Adding tooltips explaining some of the buttons would be helpful. For instance, in the Multi Shortest Path Tree result panel, the functionality of the button 'pass through S-P' is not obvious for someone who has not read the article.
3. Pressing on one of the found shortest paths selects the network nodes involved in it, but not the edges. Selecting the edges also would make the path easier to grasp.
4. Please print less information to the console. E.g. 'pass through S-P' prints 'accendo node_name_1, accendo node_name_2, etc.'; human interactome network selection prints 'HUMAN_INTERACTOME.sif, Helloooooo, HUMAN_INTERACTOME.sif'...

Changes to the article:

1. In introduction: "in Systems Biology a common methodology implies that the informational flow follows a maximum parsimony principle (see Box 3 in 5)"
  The scope of the reference is limited to network medicine instead of systems biology in general. Please, either add another reference or adjust the scope.
2. In methods: "All Pair Shortest Paths (APSP) version of the basic Dijkstra algorithm, single thread". Please, specify why single thread is used instead of multiple ones?
3. In use cases: Creating a separate subsection/paragraph for each use case, in my opinion, would improve readability.

I have read this submission. I believe that I have an appropriate level of expertise to confirm that it is of an acceptable scientific standard, however I have significant reservations, as outlined above.

# Peer review: Referee response for version 2

**DOI:** 10.5256/f1000research.8872.r13232

**Published:** 2016-05-04

**Authors:** Ankush Sharma

**Affiliations:** 1 Laboratory for Genomics,Transcriptomics and Proteomics, High Performance Computing and Networking Institute, National Research Council, Naples, Italy

The Authors have addressed all the amendments for the concerns raised by reviewers and made necessary changes in manuscript. The manuscript is fully approved for indexing.

I have read this submission. I believe that I have an appropriate level of expertise to confirm that it is of an acceptable scientific standard.

# Peer review: Referee response for version 1

**DOI:** 10.5256/f1000research.7273.r9885

**Published:** 2015-09-01

**Authors:** Ankush Sharma

**Affiliations:** 1 Laboratory for Genomics,Transcriptomics and Proteomics, High Performance Computing and Networking Institute, National Research Council, Naples, Italy

The authors describe the useful PesCa app for Cytoscape version 3.x for computing the shortest paths in networks and shortest paths connecting any two selected nodes and reconstruction of subnetworks based on maximum parsimony principle. The PesCa app scales well with efficiency in computation time on large networks. I agree with the point raised by reviewer 1 in version 1 of referee report.

Minor concern

1. Using terminology such as isolated nodes (nodes with no connection) and giant networks  is making article confusing.
2.  Maximum parsimony principle needs to be elaborated in details. and how it scores over other methods such as maximum likelihood used for  reconstruction of weighted and unweighted networks along with one illustrative example will help in better understanding of the concept.
3. The representational figure 2 for demonstrating short paths from node 1 to node 9 can be improved by coloring edges involved in short paths with different color.

I have read this submission. I believe that I have an appropriate level of expertise to confirm that it is of an acceptable scientific standard, however I have significant reservations, as outlined above.

# Peer review: Referee response for version 1

**DOI:** 10.5256/f1000research.7273.r9882

**Published:** 2015-08-24

**Authors:** Ferenc Jordán

**Affiliations:** 1 Centre for Ecological Research, Hungarian Academy of Sciences, Budapest, 1051, Hungary

The Authors present a useful Cytoscape app for calculating shortest paths in networks.

This is useful with the following comments to be considered:

I do not suggest to use the words "isolate" and "giant component" for the situation when the distance between a well-defined node a well-defined subnetworks is to calculated. In network science, there is enough confusion about certain concepts, it is better to avoid adding more. If you refer to a "selected node" and a "selected subgraph", everything is perfectly understandable and there is no confusion.

In the second paragraph (4th line) of the Introduction, you speak about "reconstructing" networks. Why not simply "constructing"?

The maximum parsimony principle may work well or may not work at all, depending on the actual problem. I do not suggest to use it as a general assumption. It would be more interesting (and a lot more useful) to dedicate a brief paragraph for this isssue: when does it make sense (network flows flow shortest paths) and when it does not (network flows do not prefer shortest paths).

In the legend of Figure 2, you mention node 6 instead of node 1.

In Figure 5, I suggest to keep nodes 8, 9 and 10 yellow but to use another colour for node 6.

Please add a bit more explanation about how to manage information about link direction, link weight and link sign in the database. Can you convert (symmetrize, binarize) and if yes, how does it happen (e.g. how to determine weight if you symmetrize AB and BA with different weights?).

What happens if a node is really isolated (degree equals zero in the original graph)? Can you perform a calculation based on the reciprocal distance matrix?

I have read this submission. I believe that I have an appropriate level of expertise to confirm that it is of an acceptable scientific standard, however I have significant reservations, as outlined above.
